# Teaching Human Poses Interactively to a Social Robot

**DOI:** 10.3390/s130912406

**Published:** 2013-09-17

**Authors:** Victor Gonzalez-Pacheco, Maria Malfaz, Fernando Fernandez, Miguel A. Salichs

**Affiliations:** 1 Robotics Lab, Systems Engineering and Automation Department, Universidad Carlos III de Madrid, Av. Universidad 30, Leganés 28911, Spain; E-Mails: mmalfaz@ing.uc3m.es (M.M.); salichs@ing.uc3m.es (M.A.S.); 2 Computer Science Department, Universidad Carlos III de Madrid, Av. Universidad 30, Leganés 28911, Spain; E-Mail: ffernand@inf.uc3m.es

**Keywords:** interactive learning, human–robot interaction, robot learning

## Abstract

The main activity of social robots is to interact with people. In order to do that, the robot must be able to understand what the user is saying or doing. Typically, this capability consists of pre-programmed behaviors or is acquired through controlled learning processes, which are executed before the social interaction begins. This paper presents a software architecture that enables a robot to learn poses in a similar way as people do. That is, hearing its teacher's explanations and acquiring new knowledge in real time. The architecture leans on two main components: an RGB-D (Red-, Green-, Blue- Depth) -based visual system, which gathers the user examples, and an Automatic Speech Recognition (ASR) system, which processes the speech describing those examples. The robot is able to naturally learn the poses the teacher is showing to it by maintaining a natural interaction with the teacher. We evaluate our system with 24 users who teach the robot a predetermined set of poses. The experimental results show that, with a few training examples, the system reaches high accuracy and robustness. This method shows how to combine data from the visual and auditory systems for the acquisition of new knowledge in a natural manner. Such a natural way of training enables robots to learn from users, even if they are not experts in robotics.

## 1. Introduction

Human Robot Interaction (HRI) is the field of research that studies how humans and robots should interact and collaborate. Humans expect robots to understand them as other people do. In this aspect, a robot must understand natural language and should be capable of establishing complex dialogues with its human partners.

However, dialogue is not only a matter of words. Most of the information that is exchanged in a conversation does not come from the phrases stated by the people engaged in this speech, but from their non-verbal messages. For instance, gestures encompass a great part of the non-verbal information. Moreover, there are also other factors that provide information, such as the postural information that a person shows to her listeners.

Gesture and pose recognition systems have been an active research field in recent years [1]. However, traditional image capture systems require the use of complex statistical models to recognize the body, making them difficult to be used in practical applications [2].

Recent technological developments are making new types of vision sensors more suitable for interactive scenarios [3]. These devices are depth cameras [3–5], which make the extraction of the human body easier than with traditional cameras. Therefore, since extracting the body is much less CPU-consuming now, it is possible to execute, in real time, algorithms that actually process the user's gestures or poses.

Specially relevant is the case of the Microsoft Kinect sensor [6], a low-cost depth camera, which offers a precision and a performance similar to high-end depth cameras, but at a cost several times lower. Together with the Kinect, several drivers and frameworks to control it have appeared. These drivers and frameworks provide direct access to a model of the user's skeleton. This model is precise enough to track the pose of the user and to recognize her gestures in real time.

Using these new vision sensors with Automatic Speech Recognition (ASR) systems enables robots to learn interactively from human examples. In this paper, we present a software architecture that enables the social robot, Maggie [7], to learn human poses using depth information coming from a Kinect camera and to process the user's voice explanations in real time. We take advantage of our robot's interaction capabilities to let it learn poses by interacting with its human teacher. In short, the user acts as a teacher, telling it in which pose she is standing. The robot is able to understand what the user is saying and fuses that information with the depth data to learn the current user pose. In such an interactive way, the robot is able to learn new knowledge through interaction with the users, and new context knowledge can be acquired incrementally in the long-term.

This document is organized as follows. Section 2 presents an overview of the related work in pose and gesture classification with depth cameras. Section 3 introduces an overview of the hardware and software systems that act as the building blocks of the developed architecture. This section describes hardware components, such as the robot, Maggie, and the Kinect camera, as well as the software modules that act as the scaffold of the project. Section 4 describes the developed software architecture and is followed by the description of the experimental validation we have carried out to validate our architecture in Section 5. Finally, Section 6 closes the paper, presenting the main contributions, broader issues and future remarks that are still open with our approach.

## 2. Related Work

### 2.1. Pose Recognition Using Depth Cameras

Depth cameras are systems that can build a 3D depth map of a scene by projecting light to that scene. The principle is similar to that of Laser Interferometry Detection and Ranging (LIDAR) scanners, with the difference being that the latter are only capable of performing a 2D scan of the scene, while depth cameras scan the whole scene at once.

Depth cameras are an attractive tool in several fields that require intense analysis of the 3D environment. Two surveys thoroughly describe the field. The first one [8] dates from 2009 and surveys the technologies and applications prior to the release of the Kinect Sensor. This sensor revolutionized the field by making available a high-resolution and high-precision technology at consumer prices. A more recent survey (2013), but more focused on algorithms for body-motion analysis, is presented in [9].

However, the idea of using depth cameras for body analysis is not recent. For example, in references [10,11] their use to locate body parts is proposed. Since then, many other works have researched gesture recognition with depth cameras [12–16]. Some of these works rely on kinematic models to track human gestures once the body is detected [17–19].

Most of these works rely on capturing only one or few parts of the body. However, recent kinematic approaches, like the one in [18], make possible the tracking of the whole body without a significant increase in CPU consumption. Schwarz *et al.* [20] propose a method to estimate the full body by transforming the foreground depth image into a point cloud. Then, they determine the centroid of this point cloud and find the primary landmarks by calculating the geodesic distance along the 3D body mesh. Shotton *et al.* [21] are the authors of the human pose estimation technology used in the Xbox. They proposed a skeleton model, where the joints are fitted to previously labeled body parts using mean shift.

Our approach focuses on teaching concepts interactively to a social robot. Therefore, rather than extracting the body and tracking it directly, it uses the available technologies and algorithms as data-sources, which will be used to enable the grounding of high-level concepts, such as the name of a certain pose. Concretely, our vision system relies on the OpenNI (NI stands for Natural Interaction) [22] libraries for body extraction and tracking. OpenNI's skeleton tracking is similar to the ones mentioned above.

### 2.2. Machine Learning in Human–Robot Interactions

Fong *et al.* present a survey [23] of the interactions between humans and social robots in which the authors stress that the main purpose of learning in social robotics is to improve the interaction experience. At the time of the survey (2003), most of the learning applications were used in robot-robot interaction. Some works addressed the issue of learning in human–robot interaction, mostly focusing on imitating human behaviors, such as motor primitives. According to the authors, learning in social robots is used for transferring skills, tasks and information to the robot. However, the authors do not mention the use of learning for transferring concepts, such as poses, that enable the robot to understand the user better.

Later, Goodrich and Schultz [24] stressed the need of robots with learning capabilities, because of the complexity and unpredictability of human behaviors. They pointed out the need of a continuous learning process, where the human can teach the robot in an *ad hoc* and incremental manner to improve the robot's perceptual ability, autonomy and its interaction capabilities. They called this process *interactive learning*, and it is carried out by natural interaction. Again, their survey only reports works that referred to learning as an instrument to improve abilities, behavior, perception and multi-robot interaction. No explicit mention was made to use learning to provide the robot with high level concepts, such as the user's pose. The same occurs in [25], which presents a survey of several Learning from Demonstration (LfD) approaches and categorizes them depending on how robots collect the learning examples and how they learn a policy from these examples.

A few works use learning to teach concepts to the robot. This is the case of [26], where the authors train a mobile robotic platform to understand concepts related to the environment in which it has to navigate. The authors use a feed forward neural network (NN) to train the robot to understand concepts, like doors or walls. They train the NN by showing it numerous images of a trash can (its destination point), labeling each photo with the distance and the orientation of the can. However, the work presented some limitations, such as the learning process lacked enough flexibility to generalize the work to other areas.

More importantly, there are some works where interactive teaching relies on the teacher's voice to acquire knowledge [27,28], but to the extent of our knowledge, most of these works rely on simple interactions, often using one or a few voice commands to describe the examples. Our work is closer to Rybski *et al.* [29], where the authors created a dialogue-based system used to teach a robot different tasks. This paper presents an approach similar to Rybski's and his colleagues, in the sense of dialogue complexity for teaching, but, in our approach, instead of only using dialogue for describing the concept to be learned, we use it to describe what the robot is seeing. We also include visual information in the learning process.

Other similar approaches are [30,31]. Both consist in the integration of visual and aural systems to teach a humanoid robot different spatial concepts. Despite that their ideas are similar to our work, there are some differences. First, the robot in [30] learns spatial concepts related to objects, while our focus is on human-pose learning. Second, the authors in [30] focus on the description of the cognitive architecture. Our approach, however, focuses on the HRI point of view. In this way, while the interaction in [30] leans on simple commands issued by the tutor, in our work, the teacher has a wide range of utterances that can be used to teach the same pose to the robot.

On the other hand, Heckmann *et al.* [31] focus on the aural system of the robot to enable it to understand the user without the need for an external microphone. Compared to our work, in [31], the robot not only learns the visual representation, but also the auditive labels. However, each new auditive label needs five to eight repetitions from the tutor to be learned by the robot. Our approach prefers to ease the interaction during the learning session. Although our grammars must be pre-written by the robot programmer, they facilitate the task of the tutor by allowing one to use many different utterances to refer to the same semantic label. This is an advantage to the tutor, who can express herself in a much more natural way.

## 3. Hardware and Software Platform

Before entering into the design of our proposed system, we introduce the different building blocks that are necessary to build our system. In this section, we describe the different hardware and software components that enable our robot to learn the poses while interacting naturally with the user.

### 3.1. The Robot, Maggie

Maggie is a robotic platform developed by the RoboticsLab team at the Carlos III University of Madrid aimed at research in social robotics and HRI.

**Figure 1.**
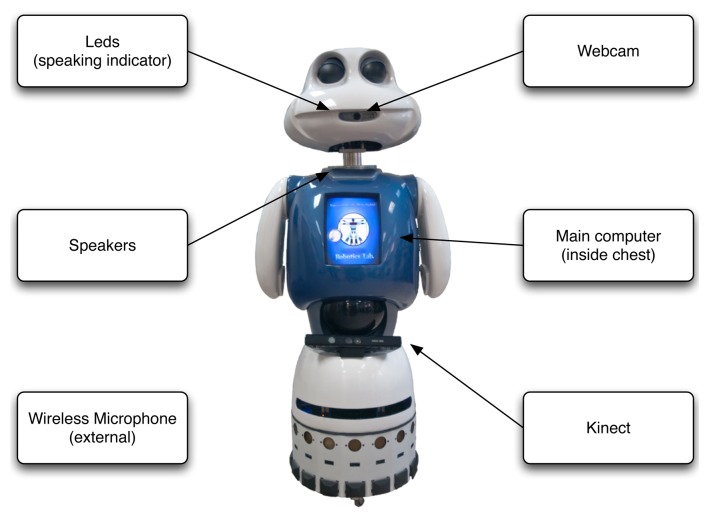
**Interaction elements of the robot, Maggie**—Among all of them, the learning system relies on the data captured by the Kinect and the wireless microphone.

The hardware components of the robot are intended to allow the robot to interact with its environment and with humans. The most relevant ones are depicted in Figure 1. Except the webcam, all of them are used in this work. A complete description of the robot can be found in [7].

### 3.2. The Kinect Vision System

The Microsoft Kinect RGB-D sensor is a peripheral that, was initially designed as a video-game controlling device for the Microsoft's X-Box Console. The sensor provides a depth resolution similar to the high-end Time-of-Flight (ToF) cameras, but at a cost several times lower. This fact has made the Kinect a widely adopted sensor in the robotics community.

This sensor retrieves the depth information using light coding technology, which consists of emitting a dot pattern onto the scene [5,32]. The Kinect is composed of one infrared (IR) emitter, responsible for emitting the light pattern onto the scene, a depth sensor, responsible for capturing the emitted pattern, and a standard RGB sensor that records the scene in visible light. The system has a spatial resolution of 3 mm in the *x*/*y* axes and of 1 cm in the *z* axis at a distance of two meters. This spatial resolution is sufficient to accomplish pose detection tasks.

### 3.3. The AD Software Architecture

Maggie's main software architecture is an implementation of the Automatic-Deliberative (AD) Architecture [33]. The basic component of the AD architecture is the *skill* [34]. A skill is the minimum module that allows the robot to execute an action, such as moving through the environment, reading the output from a laser sensor or communicating with a human.

In essence, a skill is a process that carries out computing operations and shares the results of these operations with other skills. For example, imagine a skill that is in charge of detecting obstacles using the laser readings. In this case, the main operations of the skill are: reading the laser data, deciding whether there is an obstacle or not and making this information available to other skills. The sharing mechanisms used by the skills are events (a communication mechanism that follows the *publisher*/*subscriber* paradigm described by Gamma *et al.* in [35]) or a shared memory system.

AD is implemented in the ROS (Robot Operating System) framework. ROS [36] is an open-source, meta-operating system for robots. It provides services similar to the ones provided by an operating system (OS), including hardware abstraction, low-level device control, implementation of commonly-used functionalities, inter-process communication and packet management. Additionally, it also provides tools and libraries for obtaining, building, writing and running code in a multi-computer environment.

The main concepts of ROS that are relevant to this paper are nodes and topics. The former are the minimum unit structure of the ROS architecture. They are processes that perform computation. Essentially, each AD skill is implemented as an ROS node. The latter, topics, are the communication system that enables the information exchange between nodes. They are, in fact, an implementation of the AD's events. Since AD is a conceptual architecture and ROS its implementation, in the rest of the paper, we might refer to the terms skill/node and event/topic indistinctly.

### 3.4. Description of the Voice System of the Robot

To enable the robot to communicate by voice with the user, two software tools are needed: a Text To Speech (TTS) tool to talk with the user and an Automatic Speech Recognition (ASR) tool to hear and understand what the teacher says. The first tool, TTS, is a technology that transforms written information into spoken words, that is, a TTS says any text that it receives as an input. On the contrary, an ASR transforms any human utterance, captured by the microphone of the robot, to written text, which can be understood by a computer.

AD uses commercial TTS and ASR tools provided by Loquendo [37]. This vendor provides Application Interfaces (APIs) for both TTS and ASR. These APIs are wrapped in the form of two skills: the *ETTS Skill* (Emotional Text to Speech) and the *ASR Skill* [38]. These are wrapped in the form of skills, so they permit other skills to send utterances to the *ETTS skill* and to retrieve what the user has said from the *ASR Skill* by simply using the communication mechanisms provided by AD.

#### 3.4.1. Enabling the Robot to Talk: The ETTS Skill

Maggie's speaking capabilities lean on the ETTS Skill. The ETTS Skill wraps and adds some features to the Loquendo TTS API. We chose Loquendo's TTS over others because of its superior voice synthesis quality and its greater configuration possibilities [39].

It is possible to configure several utterance parameters, such as the tone or the speed of the locution. This allowed us to create four predefined emotional states that the robot can express: calmed, nervous, happy and sad. Thanks to that, the robot produces a high quality synthetic voice that is pleasant to humans and that improves the likability of the robot. A grammar establishes a series of words or a combination of words and links them to a semantic meaning. The semantics of those words are coded into labels that can be codified as variables in a computer program. When a skill wants to make the robot talk, it simply sends an event to the ETTS Skill with the utterance to say and with the emotion parameters to express. The ETTS Skill manages Loquendo's API to produce the utterance with the appropriate desired parameters.

#### 3.4.2. Speech Recognition: The ASR Skill

To maintain dialogue with the user, the robot not only needs to speak, but also to understand what she is saying. This task is carried out by the ASR Skill, which transforms the utterances of a person into text.

The ASR skill uses predefined grammars to detect and process the user's speech [38]. An ASR grammar is a set of words and phrases linked to a semantic meaning. When the user is speaking, the robot tries to match the user's utterances to the sets of the loaded grammar. When a match is produced, the robot returns the semantic meaning of that phrase. For instance, a grammar to understand when the user is saluting the robot could be defined by the phrases: “*Hello!*”, “*Good morning*, *Maggie*”, “*Hi Maggie, how are you today?*”, *etc.* If the robot detects an utterance similar to these ones, it will interpret that the user is greeting it, so it can act accordingly.

A more formal definition of a grammar is the following: A grammar, *G*, is defined by the tuple, *G* = (*U*, *s*), where *U* is a set of utterances and *s* is the semantic meaning associated with *U*. The robot can load and unload different grammars in real time, to adapt itself to different HRI contexts.

Note that there are several utterances *U* = {*u*_1_, *u*_2_, …, *u_n_*} that can be associated with a single semantic meaning, *s_i_*. Each utterance, *u_i_*, might be composed of single words, phrases or any combination of them. For instance, the semantic meaning *s_1_* = *raining* might be composed of the utterances:

*u*_1_ = [*] *raining* [*]
*u*_2_ = [*] *[take the]* *umbrella* [*]

where the asterisk, *, acts as a wildcard to indicate any word or set of words and the square brackets indicate that the elements enclosed by them are optional. Therefore, utterances like “*It seems it is raining*.” and “*Take the umbrella before going out*.” trigger *s*_1_.

In the case of pose learning, we have defined two grammars, *G_p_*, which defines the different poses the users can adopt while teaching the robot, and *G_c_*, which defines the interaction commands during the learning process. These grammars are further defined, later, in Section 4.1.2.

#### 4. Learning Architecture

**Figure 2.**
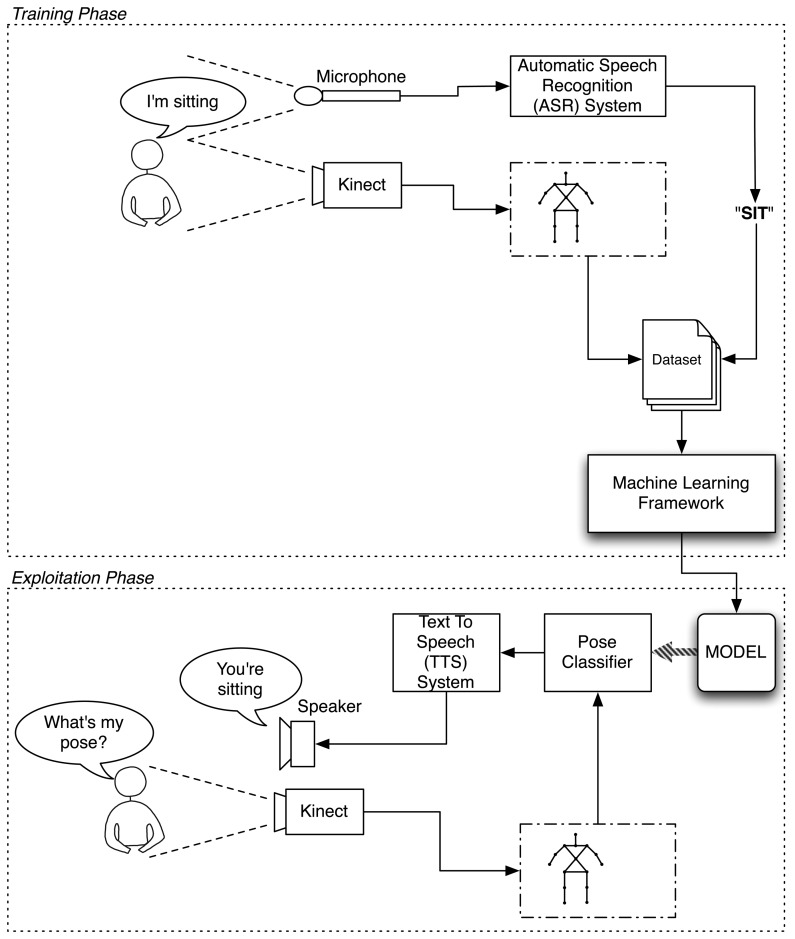
System Overview—The upper part of the diagram shows the training phase, where the user teaches the robot, by verbal commands, which are the poses that the robot must learn. The lower part of the diagram depicts the exploitation phase, in which the robot loads the learned model and tells the user's current pose by its voice system.

This section describes all the modules that have been built to enable the robot to learn poses from the human by interacting with one. Figure 2 depicts the general scheme of the built architecture. The diagram separates the training and the exploitation phases. The upper part represents the training phase, where the user teaches the system to recognize certain poses. The lower part of the figure represents the exploitation phase, in which the robot uses what it has learned to discern in which pose the user is standing.

In the training phase, the robot uses two sensory systems. The first one is its Kinect-based RGB-D vision system. With it, the robot acquires the figure of the user separated from the background and processes it to extract a kinematic model of the user's skeleton using OpenNI's algorithms. The second input is the Automatic Speech Recognition Skill (ASR), which allows the robot to process the words said by the user and converts them into text strings.

The data of these sensors is fused to create a learning instance defined by:

- **The pose of the user**, defined by the configuration of the joints of the kinematic model of the user.
- **A label identifying this pose**, defined by the text string captured by the auditive system of the robot.

While the user is training the robot, the Kinect keeps acquiring data from the user at 30 frames per second (FPS). These data are labeled according to the label given by the user and stored in a dataset. Once the training session has finished, the dataset is processed by the Weka machine learning framework [40], which builds a model establishing the relations between the poses and their associated labels. That is, the learned model establishes the rules that define when a determined pose is associated with a certain label.

In the exploitation phase, the robot continues receiving snapshots of the skeleton model at every frame. However, this time, it does not receive the auditive input telling it what the user's pose is. Instead, the robot has to predict it using what it has learned from the user. For this purpose, it loads the incoming Kinect's data to the learned model, which evaluates it and returns the guessed label corresponding to that pose.

To provide feedback, the robot says the pose in which it believes the user is standing.

This is done by providing the guessed pose label to the ETTS skill of the robot, which is in charge of transforming this label to an utterance that the user can understand.

### 4.1. Data Acquisition and Preparation

This section enters more detail into how the data is captured and processed before it is fed to the learning system. First, it describes the robot's vision system and, later, its auditive system.

#### 4.1.1. Processing Visual Data

The Kinect data provides raw depth data in the form of a 3D point cloud. This point cloud has to be processed before it is fed to the learning system. The preprocessing is carried out by an external library, named OpenNI (NI stands for Natural Interaction).

**Figure 3.**
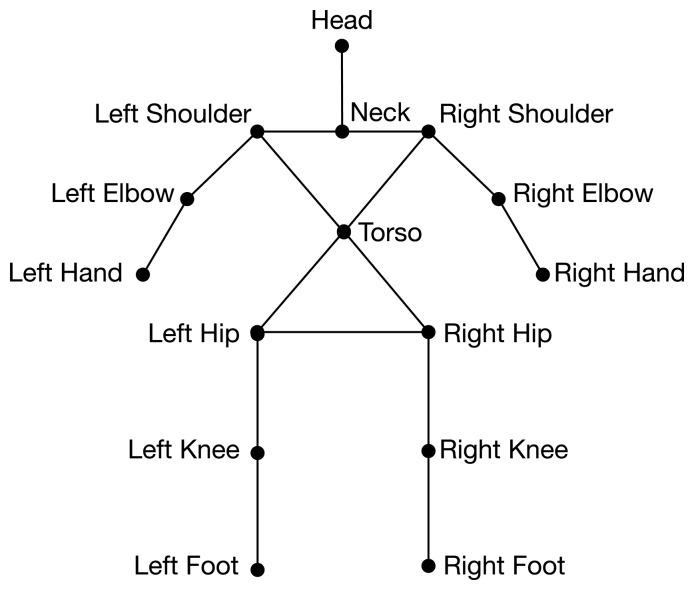
**OpenNI's kinematic model of the human body**—OpenNI (NI stands for Natural Interaction) algorithms are able to create and track a kinematic model of the human body.

OpenNI provides tools to extract the user's body from the background and to build a kinematic model from it. This kinematic model consists of a skeleton, as shown in Figure 3. OpenNI's algorithms provide the positions and orientations of these joints at a frame rate of up to 30 FPS (frames per second).

The skeleton model is the model used in our system to feed our pose detection system. In other words, the information that is provided to the learning framework comes from the output of OpenNI's skeleton extraction algorithms.

This model contains the data that is going to be used in our learning system. The data of each skeleton instance (*S*) is composed of 15 joints represented as:

(1) S=(t,u,J)

where *t* is the time-stamp of the data frame, *u* is the user identification (Here, the user identification refers to the user being identified by the openNI framework. It is a value between one and four, and it serves only in the case that more than one user is being tracked by the openNI's skeletonization algorithm.) and *J* represents the joint set from the user's skeletonized model depicted in Figure 3:

(2) $$J=(j_{1},j_{2},\ldots ,j_{15})$$

These joints have the following parameters:

(3) $$j_{i}=(x,y,z,q_{x},q_{y},q_{z},q_{w},C)$$

where *x*, *y*, *z* represent the position of the joint in *R*^3^, *q_x_*, *q_y_*, *q_z_*, *q_w_* represent the orientation of the joint as a quaternion, and *C* is the binary confidence of the values of both position and orientation of the joint as provided by openNI. *t*, *u* and *C* were not used to learn. However, they provide useful control and state information that help to detect and recover from errors or to maintain lively interaction. For instance, if the robot detects that the user has been lost from its line of sight for a long period of time, it can ask for assistance or notify the user that it is not seeing her.

#### 4.1.2. Processing Verbal Data

When the user starts training the robot, she executes two tasks. First, she stands in the pose that she wants to show the robot, and second, she tells it the name of that pose. From the robot's point of view, firstly, it has to “see” the teacher's pose, and secondly, it has to understand what she is telling it. Since the user indicates her pose by voice, the robot needs to process these pose names or labels using an auditive module. This module is the ASR Skill, previously described.

As described in Section 3.4.2., the ASR skill needs a grammar to process the user speech. In the case of pose learning, the robot needs a grammar to understand what the user says during the training session. Therefore, we have built a grammar, *G_p_*, that enables the robot to detect up to 18 pose definitions by combining different semantics in three different categories:

(4) $$G_{p}=(s_{p},s_{a},s_{d})$$

where *s_p_*, *s_a_*, *s_d_* are three semantic meanings of the user's speech:

1. **Posture Semantics**, s_p_: This can take one of the following values:
  $$s_{p}=\{\text{sit},\text{stand}\}$$
  where:
  (a) *sit* defines that the user is sitting on a chair.
  (b) *stand.* defines that the user is standing in front of the robot.
2. **Action Semantics**, **s_a_**: This can take one of the following values:
  $$s_{a}=\{\text{turned},\text{looking},\text{pointing}\}$$
  where:
  (a) *turned* defines that the user is turned (oriented her body) to the direction specified in *s_d_*.
  (b) *looking* defines that the user has oriented her head to the direction specified in *s_d_*.
  (c) *pointing* defines that the user is pointing in the direction specified in *s_d_*.
3. **Direction Semantics**, **s_d_**: This can take one of the following values:
  $$s_{d}=\{\text{left},\text{forward},\text{right}\}$$
  where:
  (a) *left* defines that the action defined in *s_a_* is carried out to the user's left side. For example, if the trainer is pointing (*s_a_* = *pointing*), she is doing it to her own left.
  (b) *forward* defines that the action defined in *s_a_* is carried out toward the user's front. For instance, if the trainer is pointing (*s_a_* = *pointing*), she is doing it toward her own front.
  (c) *right* defines that the action defined in *s_a_* is carried out to the user's right. For instance, if the trainer is pointing (*s_a_* = *pointing*), she is doing it to her own right.

A complete list of the semantic meanings that *G_p_* can understand is shown in Table 1.

When speech is detected, the ASR Skill evaluates this speech against *G_p_*. If the evaluation produces a valid result, the ASR tags the speech with a label, $l_{\left(i\right)}^{p}\in G_{p}$ where *i* is the *i^th^* label. Note that *i* ∈ (1, 18), since the cardinality of *G_p_* is defined by all the possible combinations of its semantics. A grammar only is considered valid if the three semantics, *s_p_*, *s_a_* and *s_d_*, have been detected in the speech. Some label examples might be $l_{p}^{\left(1\right)}=\left(\text{sit},\text{looking},\text{left}\right)$, which indicates that the user is sitting and looking to her or left, or $l_{p}^{\left(2\right)}=\left(\text{stand},\text{turned},\text{forward}\right)$, which indicates that the user is standing and turned toward the robot.

Note that despite these being the semantic meanings that indicate what the user has said, it is possible to arrive at these semantics with different words or phrases. That is, each semantic meaning of *G_p_* has between three and five utterances associated with it, as described in Section 3.4.2. For instance, phrases, such as “*I'm sitting looking to the right*” or “*I'm in a chair, looking towards my right*” would produce the same semantic meaning: $l_{p}^{\left(1\right)}=(\text{sit},\text{looking},\text{right})$

The output of the ASR Skill is sent to another skill called the *Pose Labeler Skill*. This skill first processes the results from the ASR to detect if the label told by the user is valid and, then, formats these data properly, so it can be passed to a third skill, the *Pose Trainer Skill*, which is in charge of the learning system itself.

In addition to the grammar, *G_p_*, shown above, the *Pose Labeler Skill* uses an additional control grammar, *G_c_*, which has no direct relation with the poses. This grammar acts as a control layer that allows the user to control some aspects of the training phase. The grammar, *G_c_*, is defined as *G_c_* = {*s_c_*}, where *s_c_* is its only semantic content defined by only two values: *s_c_* = {*change*, *stop*}. These values have the following meanings:

- *change*: used to allow the classifier to discriminate the transitions between two poses. It is used before she changes her pose. Examples of sentences that trigger this value can be: “Let's learn the next pose”, or “I’m going to teach you another pose, Maggie”.
- *stop*: used to end the training process. When the user says she wants to finish the training process, the ASR builds this semantic to allow the *Pose Labeler Skill* to end. Some examples of sentences that trigger this semantic value are: “So we have finished.” and “Let's stop for a while.”

As can be seen, the ASR Skill of the robot enables it to understand natural language. This makes the learning session much more natural and pleasant for the user, who can train the robot even without being a robotics expert.

### 4.2. Learning from Gathered Data

Once the visual and verbal data have been preprocessed, they are delivered to the *Pose Trainer* Skill, which is in charge of learning from these data. To train our system, the *Pose Trainer* skill stores each joint set, *J*, that has been received from the vision system in a training instance, *I*, and it tags it with a label, *l^i^* ∈ *G_p_*, described in Section 4.1.2.:

(5) $$I^{j}=\left(J^{j},l_{p}^{i}\right)$$

where *j* ∈ (1, *m*) and *m* are the number of training examples of the session. Each training instance, *I^j^*, represents a user pose and is defined by a joint set, *J^j^*, and a label, $l_{p}^{i}$. Note that *j* ∈ (1, *m*), while *i* ∈ (1, *n*), which, in the case of *G_p_*, is 18 (see Section 4.1.2.). In short, this means that the user can show different examples, *J^j^*, of the same pose, $l_{p}^{i}$.

During the training process, the user shows m different training instances to the robot, which are stored in the dataset,*D*:

(6) $$D=\left\{I^{\left(1\right)},\ldots ,I^{\left(m\right)}\right\}$$

The dataset size (the value of *m*), can vary because the training process continues until the user decides to stop it by telling it to the robot. In that case, the ASR skill will return a label, *l_c_* = *stop*, indicating to the system that the user has stopped the training process.

Our learning system is built on top of the Weka Framework [40], a widely used open-source software, which allows us to use several algorithms to build our model. Therefore, with the dataset already completed, the *Pose Trainer* Skill calls the Weka API in order to build a model from the dataset. This model can be represented by the set of associations of joint sets, *J*, and its corresponding pose labels, *l^i^*:

(7) $$M=\left\{J\to l^{i}\in G_{p}\right\}$$

This model represents the poses that the robot has learned from the user. The quality of the learned poses, *i.e.*, how well they are able to generalize to other situations depends, mainly, on the number and the quality of the examples that the user has provided to the system.

### 4.3. Using What the Robot Has Learned

The model, M, is what the robot loads during the exploitation phase in order to guess the user's pose. To do so, a skill called the *Pose Classifier* Skill loads the learned model and starts to feed it with data coming from the Kinect. For each received joint set, *J*, *M* returns the label, *l^i^*, which it believes better corresponds to that pose.

We have created a interactive test that allows us to know how well the robot has learned. In this test, the robot tells which pose the user is standing in when it is commanded to do so. This skill is called the *Pose Teller* Skill. The *Pose Teller's* main functionality is to receive the estimated label from the *Pose Classifier* Skill, to translate it to a human-readable text string and to send this string to the ETTS Skill, which is in charge of saying this text to the user.

The translation from a label, *l^i^*, to a string that a person can understand is the inverse process that was carried out in the ASR Skill, where the user speech was processed according to a grammar and its semantic meanings extracted. For instance, the label, *l* = {*sit*, *looking*, *right*}, may be transformed into a string, like: “*You're sitting looking to your right*”.

## 5. Experiments

### 5.1. Scenario Description

To test the system, we prepared a scenario in which 24 students taught the robot 3 different sets of poses:

- First, *P*_1_ consisted of teaching the robot if the trainer was turned to his/her own left, right or if he/she was turned toward the robot.
- Second, *P*_2_ consisted of teaching the robot if the trainer was looking to his/her own left, right or forward.
- Third, *P*_3_ consisted of teaching the robot if the trainer was pointing at his/her own left, right or forward

**Figure 4.**
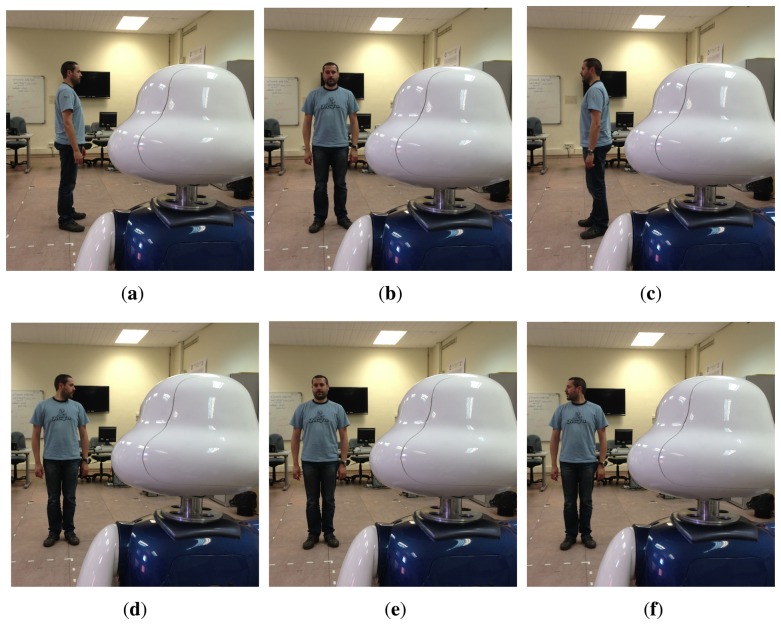

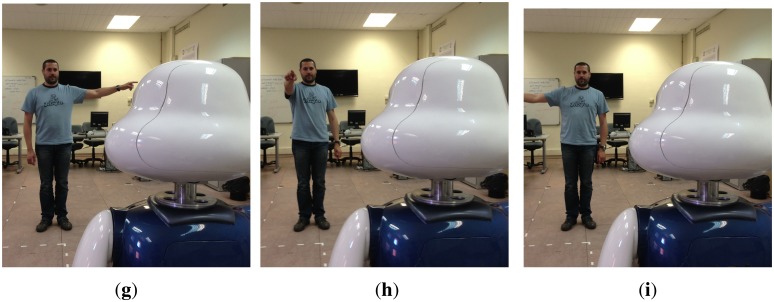
Examples of poses that the users taught to the robot. (**a**) Turned Left; (**b**) turned forward; (**c**) turned right; (**d**) looking left; (**e**) looking forward; (**f**) looking right; (**g**) pointing left; (**h**) pointing forward; (**i**) pointing right.

An example of the poses that were taught to the robot is depicted in Figure 4. We used our grammar, *G_p_*, to evaluate if the robot was able to learn interactively by carrying out a natural conversation with the users.

**Figure 5.**
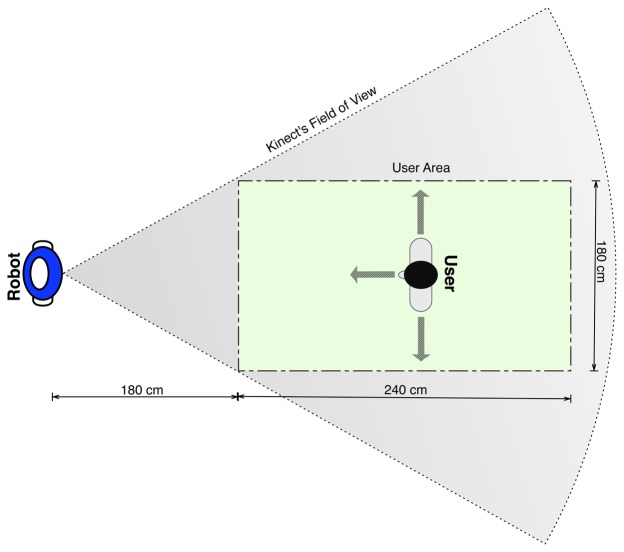
**Scenario of the experiment**—The cone represents the field of view of the Kinect sensor. The user was allowed to move inside the rectangle, as long as she always kept in the same pose while moving.

The experiment scenario is depicted in Figure 5. The colored rectangle shows the area in which the users had to remain during the training phase. It was drawn in the ground to enable the users to see whether they were inside of it. The motivation for setting a limit to the experimental area was to ensure that the user stayed inside the Kinect's field of view during the training process. However, the users were allowed to move wherever they preferred, as long as they stayed inside the rectangle while recording the poses. Finally, they were told to warn the robot before changing their pose, to prevent the recording of transitional poses.

To avoid possible confusion between the user's utterances, we raised the ASR minimum confidence to 50%. That is, the confidence of an utterance belonging to a certain semantic value, *s_i_*, had to be higher than this minimum value. This threshold was enough to avoid misclassification of semantic concepts. In the cases when the user's speech did not obtain this minimum confidence, the robot asked the user to repeat the utterance. However, these situations were occasional, and in the worst case, the user only needed a few attempts until she was able to make the robot understand her.

### 5.2. Method

Before the experiment started, the experimenter explained to the users the experimental procedure. It was indicated to them that they could ask the experimenter any questions related to the poses or the grammar commands whenever they wanted. The user has the initiative during the training process, being able to start, pause and finish the process at any moment. The experimental procedure consisted of the following steps: First, the user stands in front of the robot. Once the robot tells her that it is ready to start, she can begin the training when she considers it appropriate. For the recording of each pose, the user was told to, first, stand at a particular pose and, then, tell the robot which label defines this pose. Prior to changing to another pose, the user had to ask the robot to stop recording this pose. Once the robot tells the user that it is ready for recording the next pose, she is free to move to the next pose and start the process again. The user finishes the teaching session by issuing the “*stop*” command of the control grammar.

The users were encouraged to start with the left poses, to continue with the front poses and to finish each round with the right poses. The reason for keeping the order is because, despite the order in which the robot learns not being relevant, this helped us to analyze the data after the training. For instance, we found that three users tagged their pose to the left when they were looking (one case) and pointing (two cases) to their right. Since they were told to produce the poses in order from left to right, we assumed that they made a mistake when labeling.

We stored three datasets (*D*_1,2,3_) per user, each one corresponding to the training of a set of poses, *P_i_*, as described before. Each dataset consisted of a set of training instances, as described in Equation (5). Although these poses represent a reduced pool of poses, we believe that they are representative of how the robot can learn by interacting with the user. Note that our focus here is not on the learning problem itself, but on the capability of the robot to learn by using natural interaction with the user.

### 5.3. Results

To train the system, all the datasets corresponding to the same pose, *P_i_*, were combined into a bigger dataset, *D_i_*, that was used to create a model of the learned concept. We evaluated our system using a modification of the cross validation (CV) method. Concretely, we used a 10 times twelve-fold CV, where each fold corresponded to all the instances of two users. This evaluation method is a slight variation of the CV evaluation method [41].

We performed the evaluation in such a way because we noticed that most of the examples shown by the same user had a strong correlation. On average, each user recorded 179 examples for *D_1_*, 156 for *D_2_* and 160 for *D_3_*. This led to having several instances of the same pose that were very similar to each other.

Because of that correlation, in case we had performed a typical CV rather than our modified one, the evaluation of the system might have led us, erroneously, to conclude that the system generalizes better than it actually does. This effect is caused because, in a normal CV, the dataset is split into several folds of the same size. The problem comes if one of these folds divides the examples provided by the same user, putting some of them in different folds. When one of these folds is used to test the training set, the results will be far better than expected, because we are evaluating our training dataset with similar data to the one we used to train the system. Therefore, to avoid having some instances of the same user in training and testing sets, we decided to force the CV to create the foldings by the number of users instead of splitting the dataset into a fixed percentage of instances.

In summary, we realized that the difficulty of generalization comes from between different users, not among learning instances of the same user. Thus, to evaluate how capable our system is at generalizing what it has learned, we have to perform the evaluation against examples coming from other users who have not trained the system.

We evaluated our system using four different algorithms: J48 [42], Naive Bayes [43], Random Forests [44] and SMOs (Sequential Minimum Optimization) [45]. The detailed performance of each algorithm is shown in Table 2. Table 2a shows the performance of the four algorithms on dataset *D*_1_ (*Turned*), Table 2b depicts the performance on dataset *D*_2_ (*Looking*) and Table 2c depicts the performance on dataset *D_3_* (*Pointing*).

The tables show different performance metrics (accuracy, true positive rate, false positive rate, precision, recall and F-measure) for the four algorithms that we used to test our system. The results are an average of all the CV runs described above. We provide, as well, the standard deviation and the 95% confidence interval (95% CI) for each one of the metrics.

The 95% CI is obtained from:

(8) 95%CI=M±1.96SE

where *M* is the mean and *SE* is the standard error obtained from:

(9) $$\text{SE}=\frac{\text{SD}}{\sqrt{N}}$$

where *SD* is the standard deviation and *N* is the number of runs of the cross validation process, which is, in this case *N* = 120.

shows the learning curves of the algorithms on datasets *D*_1_ (*Turned*), *D*_2_ (*Looking*) and *D*_3_ (*Pointing*), respectively. The curves represent the average accuracy and the 95% CI of the system when trained with 2, 4, 6, …, 22 users. The values of the training with 22 users are the same as the ones reflected in Table 2. Note that the maximum number of training users is 22 instead of 24, because the CV forces that in each run there will be two users for the evaluation of the training set of that run.

**Figure 6.**
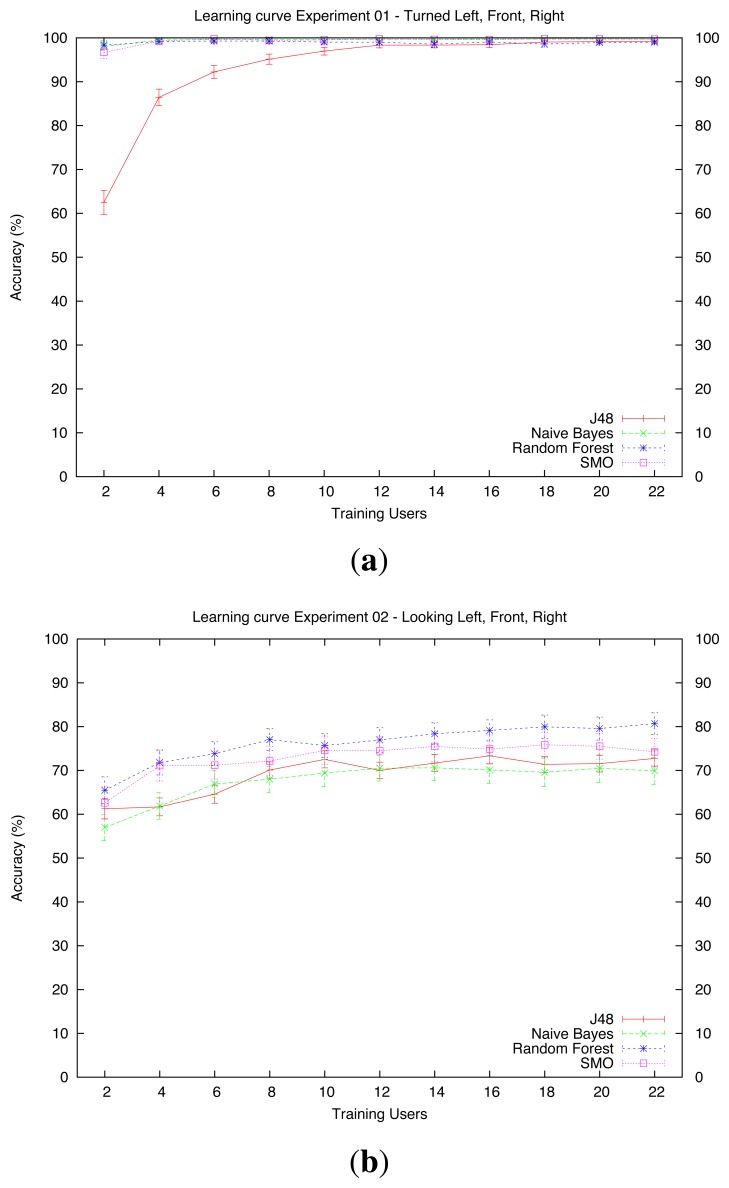

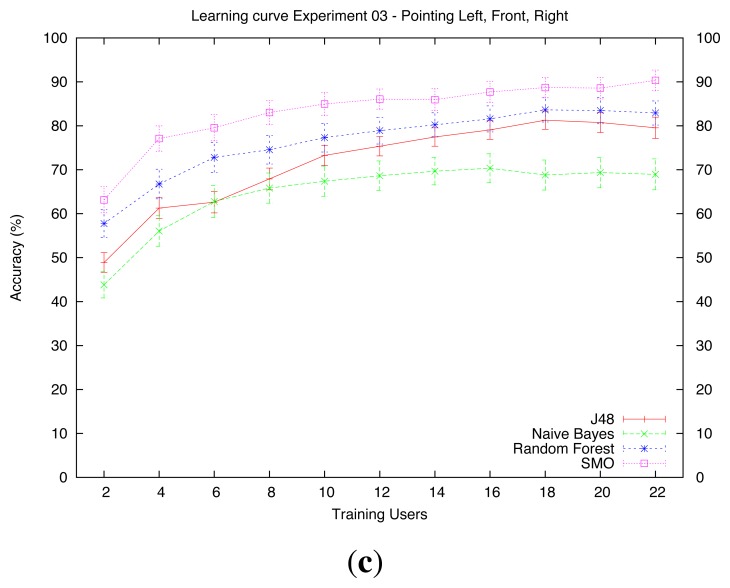
Learning curves for the three datasets. (**a**) Dataset *D*_1_ (turned left, front, right); (**b**) dataset *D*_2_ (looking left, front, right); (**c**) dataset *D*_3_ (pointing left, front, right).

- In dataset *D*_1_ (*turned*,Figure 6a), all the algorithms scored nearly 100% accuracy with relatively few training users. The only exception to this is the J48 algorithm, which needed 12 users to reach the performance of the other algorithms.
- In dataset *D*_2_ (*Looking*, Figure 6b), all the algorithms showed the worst performance. We believe that this might have been because the orientation of the head is important for discerning where the user is looking. Unfortunately, the OpenNI algorithms provide less precise data for the head than for other joints; therefore, since the learning system had to deal with inaccurate data, its performance decreased.
  There is no clear difference between algorithms if they are trained with few examples. With only examples from one user, their performance ranges from nearly 60% by Naive Bayes to 65% by Random Forests. When the number of users is increased to 12, Random Forests performs better than J48 and Naive Bayes. Finally, when we have 22 users for training, Random Forests stands above the rest, achieving an 80% accuracy (see Table 2b).
  In this dataset, all the algorithms slightly increase their performance when adding more users to training. Although this increment is not significant from 12 users thereafter, we hypothesize that adding more users to *D*_2_ might help to increase the overall performance of the system.
- Dataset *D*_3_ (*Pointing*, Figure 6c) is where we found the biggest differences among algorithms. Here, Naive Bayes showed the lowest performance. With only one training user, it barely reached a 45% accuracy whereas SMO nearly scored 65%. With 22 users, Naive Bayes showed just a 69% accuracy. In this case, SMO was the algorithm that showed the best performance for this dataset, with nearly 86% accuracy with 12 training users and roughly 90% with 22 users. Similarly to the other datasets, none of the algorithms showed a significant improvement when increasing the number of training users from 12 to 22. However, like in dataset *D*_2_, we believe that adding more users might increase the performance, especially in the cases of the J48, the Random Forests and the SMO algorithms.

### 5.4. Discussion

The presented results show that it is possible to learn poses from examples provided by the users in an interactive way. Nevertheless, Figure 6 indicates that the examples provided by one single user are not enough to generalize the learned concepts to other users. On the other hand, the system needed only 12 users to achieve good classifying results.

**Figure 7.**
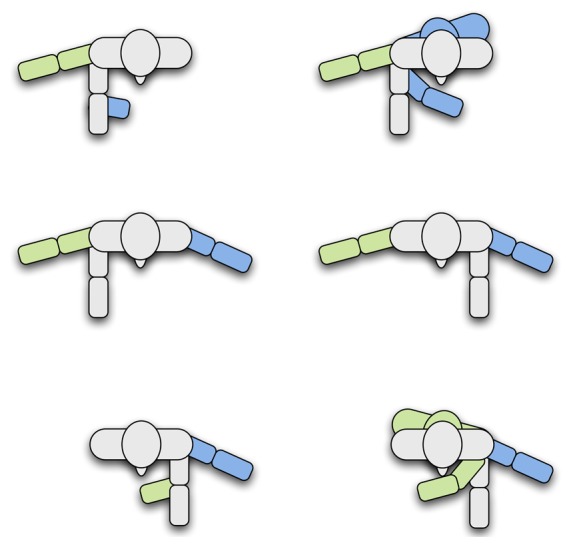
Examples of how different users pointed during the training for *D*_3_.

In general terms, during the training, we observed a great variability between the poses that each user taught the robot in datasets *D*_2_ (*looking*) and *D*_3_ (*pointing*). That is, when the robot was learning a pose, the examples shown by each user differed considerably. This effect was especially relevant in dataset *D*_3_ (*pointing*), where some users used their right hand to point, while others, their left hand. Even more, in some cases, some users used their right hand to point to their right and their front, but changed to the left hand when pointing to their left (see Figure 7). In fact, we also observed some cases in which the users looked to the direction where they were pointing, while others looked to the robot instead.

In the case of dataset *D*_2_ (*Looking*), we observed less differences, but still significant ones. Here, some users exemplified the looking poses by only turning their heads to the left or right, while others slightly turned their torso and waist, as well.

This differences between users is what may produce lower results when the robot is trained only with a few users. As it gathers examples from more users, it discovers new ways of how each pose is executed and, therefore, improves its classification accuracy.

We consider this variability one of the key justifications of our natural learning system, since it enables the robot to learn by directly asking the user, who does not need to be an expert in robotics to teach it.

Despite the solution to this variability seeming to obtain examples from many users, in some cases, it might be difficult to obtain examples from more users. For instance, the user might not have the time to train the robot. For that reason, it might be interesting to improve the generalization capabilities of the learning system. In such a way, active learning techniques, where the robot actively asks the user for information regarding the poses, might accelerate the robot's learning speed.

To conclude the discussion, we must stress that forcing the users to stay inside a fixed area during the training phase might reduce the naturalness and realism of the scenario. This was maintained by ensuring that the user joints were inside the field of view of the Kinect during the training process. However, as a consequence, most users tended to place themselves near the geometric center of the area and barely moved from there. We are currently considering the possibility of a more natural environment to allow scenes where the user might be located in some positions in which the robot might not see some of the joints of the user. On the other hand, we plan to allow the robot to track the user by moving itself, so it can adapt to the changing conditions of the scene, such as the user standing closer to or further from the robot, *etc.*

## 6. Conclusions

This paper presented a system to endow a social robot with the capacity to learn interactively by maintaining a natural conversation with its human teacher. The natural interaction is achieved using a grammar-based ASR, whose aim is to recognize different sentences and to extract their semantic meaning. Using the semantics as labels of the concept being learned, the robot is able to understand users that are not robotic experts.

Our system has been tested in the application of pose recognition, in which the robot learns the poses adopted by the teacher, listening her explanations. Our experiment consisted of 24 non-robotics experts training the robot nine different poses in three training exercises. We evaluated our system by comparing four learning algorithms, achieving satisfactory results in all of them for the three exercises.

A robot with interactive learning capabilities can adapt rapidly to different situations, since the user can train it *ad hoc* for that situation. Moreover, since the robot is capable of establishing natural interactions, the teacher does not need any expertise in robotics.

Despite the promising results, our system still presents a major limitation. The maximum number of poses it can learn is limited by the number of semantics coded into the ASR's grammar. Moreover, these grammars are pre-written in a text file by the robot programmer. However, we already started working on an extension to our system, targeted at solving this limitation. This extension consists of combining a grammar-based ASR with statistical language models. Combined, the user will be able to add new semantics to the grammar that will be used to label the learned concept, as well.

Additionally, our work leaves other paths open for exploration. Firstly, from the HRI point of view, this paper has focused on the HRI from the robot's point of view. It remains to study how users perceive what the robot has learned and how this fact changes their relation and their expectations towards it. Even more, understanding what the user thinks about the learning process might lead to better training scenarios that would end in robots that learn better from the users. Secondly, this work opens the door for building a continuous learning framework, where the robot actively seeks for new examples and asks questions of its teacher about the concepts being learned. Thirdly, with only a few minor modifications, this learning system can be extended to other applications, such as gesture learning, activity learning or the interactive learning of new objects.

## Figures and Tables

**Table 1.** Semantic values of *G_p_*. In order to be considered a valid sentence of *G_p_*, the sentence must include one semantic value from each column. If the received sentence is valid for *G_p_*, then the sentence is defining a pose. Note that *G_p_* defines the 18 poses (2 × 3 × 3) that the robot can understand and, therefore, learn.

s_p_	s_a_	s_d_
Sit	Turned	Left
Stand	Looking	Front
	Pointing	Right

**Table 2.** Learning performance for the three datasets. Note: TP = True positive; FP = False Positive. **(a)** Learning performance for dataset *D*_1_ (*turned: left, forward, right*); **(b)** learning performance for dataset *D*_2_ (*looking: left, forward, right*); **(c)** learning performance for dataset *D*_3_ (*pointing: left, forward, right*). SMO, Sequential Minimum Optimization; SD, standard deviation; CI, confidence interval.

	**J48**			**Naive Bayes**			**Random Forests**			**SMO**		
	**Mean**	**SD**	**CI 95%**	**Mean**	**SD**	**CI 95%**	**Mean**	**SD**	**CI 95%**	**Mean**	**SD**	**CI 95%**
**Accuracy**	0.991	0.029	0.003	0.997	0.009	0.001	0.990	0.026	0.002	0.997	0.010	0.001
**TP Rate**	0.991	0.029	0.003	0.997	0.009	0.001	0.990	0.026	0.002	0.997	0.010	0.001
**FP Rate**	0.007	0.025	0.002	0.001	0.003	0.000	0.007	0.020	0.002	0.002	0.008	0.001
**Precision**	0.993	0.023	0.002	0.998	0.008	0.001	0.992	0.021	0.002	0.997	0.009	0.001
**Recall**	0.991	0.029	0.003	0.997	0.009	0.001	0.990	0.026	0.002	0.997	0.010	0.001
**F-measure**	0.991	0.031	0.003	0.997	0.009	0.001	0.990	0.027	0.002	0.997	0.011	0.001

**(a)**												

	**J48**			**Naive Bayes**			**Random Forests**			**SMO**		
	**Mean**	**SD**	**CI 95%**	**Mean**	**SD**	**CI 95%**	**Mean**	**SD**	**CI 95%**	**Mean**	**SD**	**Cl 95%**
**Accuracy**	0.727	0.151	0.014	0.699	0.173	0.016	0.807	0.138	0.013	0.742	0.171	0.016
**TP Rate**	0.727	0.151	0.014	0.699	0.173	0.016	0.807	0.138	0.013	0.742	0.171	0.016
**FP Rate**	0.129	0.075	0.007	0.130	0.072	0.007	0.089	0.061	0.006	0.129	0.081	0.007
**Precision**	0.774	0.137	0.013	0.794	0.133	0.012	0.848	0.110	0.010	0.776	0.179	0.016
**Recall**	0.727	0.151	0.014	0.699	0.173	0.016	0.807	0.138	0.013	0.742	0.171	0.016
**F-measure**	0.712	0.158	0.014	0.688	0.184	0.017	0.801	0.143	0.013	0.723	0.190	0.017

**(b)**												

	**J48**			**Naive Bayes**			**Random Forests**			**SMO**		
	**Mean**	**SD**	**CI 95%**	**Mean**	**SD**	**CI 95%**	**Mean**	**SD**	**CI 95%**	**Mean**	**SD**	**CI 95%**

**Accuracy**	0.795	0.193	0.018	0.690	0.198	0.018	0.829	0.153	0.014	0.903	0.131	0.012
**TP Rate**	0.795	0.193	0.018	0.690	0.198	0.018	0.829	0.153	0.014	0.903	0.131	0.012
**FP Rate**	0.093	0.087	0.008	0.141	0.081	0.007	0.084	0.077	0.007	0.054	0.075	0.007
**Precision**	0.863	0.152	0.014	0.714	0.234	0.021	0.878	0.114	0.010	0.922	0.116	0.011
**Recall**	0.795	0.193	0.018	0.690	0.198	0.018	0.829	0.153	0.014	0.903	0.131	0.012
**F-measure**	0.785	0.205	0.019	0.656	0.230	0.021	0.823	0.161	0.015	0.899	0.142	0.013

**(c)**												
